# Supplementary material for: Giant spin-to-charge conversion at an all-epitaxial single-crystal-oxide Rashba interface with a strongly correlated metal interlayer
Source: Nat Commun. 2022 Sep 26;13:5631. doi: 10.1038/s41467-022-33350-5 (PMC9512910; doi:10.1038/s41467-022-33350-5)
Supplement: Supplementary file 1 — Supplementary Information [file 41467_2022_33350_MOESM1_ESM.pdf]

Supplementary Information

Giant spin-to-charge conversion at an all-epitaxial single-crystal-oxide Rashba interface with a strongly correlated metal interlayer

Shingo Kaneta-Takada[1*], Miho Kitamura[2], Shoma Arai[1], Takuma Arai[1], Ryo Okano[1], Le Duc Anh[1,3], Tatsuro Endo[1], Koji Horiba[2], Hiroshi Kumigashira[2,4], Masaki Kobayashi[1,5], Munetoshi Seki[1,5], Hitoshi Tabata[1,5], Masaaki Tanaka[1,5**] and Shinobu Ohya[1, 5***]

[1]Department of Electrical Engineering and Information Systems, The University of Tokyo, 7-3-1 Hongo, Bunkyo-ku, Tokyo 113-8656, Japan.

[2]Photon Factory, Institute of Materials Structure Science, High Energy Accelerator Research Organization (KEK), 1-1 Oho, Tsukuba, Ibaraki 305-0801, Japan.

[3]PRESTO, Japan Science and Technology Agency, 4-1-8 Honcho, Kawaguchi, Saitama 332-0012, Japan.

[4]Institute of Multidisciplinary Research for Advanced Materials (IMRAM), Tohoku University, Sendai, Miyagi 980–8577, Japan.

[5]Center for Spintronics Research Network (CSRN), The University of Tokyo, 7-3-1 Hongo, Bunkyo-ku, Tokyo 113-8656, Japan.

E-mail: *skaneta@cryst.t.u-tokyo.ac.jp,
**masaaki@ee.t.u-tokyo.ac.jp,
***ohya@cryst.t.u-toky.ac.jp

Supplementary Note 1: Correlated spin transport in $LaTiO_{3+\delta}$

In principle, AlO_x and $LaAlO_3$ (LAO), which are normal insulators, hamper the direct transport of a spin current. On the other hand, $LaTiO_{3+\delta}$ (LTO) is metallic. Thus, it is desirable for the transport of the spin current. Furthermore, unlike ordinary metals, in which itinerant *s*,*p* carriers dominate the transport, LTO has only up/down-spin electrons of the relatively localized *d* orbital on a Ti site at the Fermi level (E_F) (Supplementary Fig. 1). The down/up-spin states exist far above the Fermi level due to the strong on-site Coulomb repulsion of the strongly correlated system. Therefore, one may expect significant suppression of spin scattering during the spin-current transport.

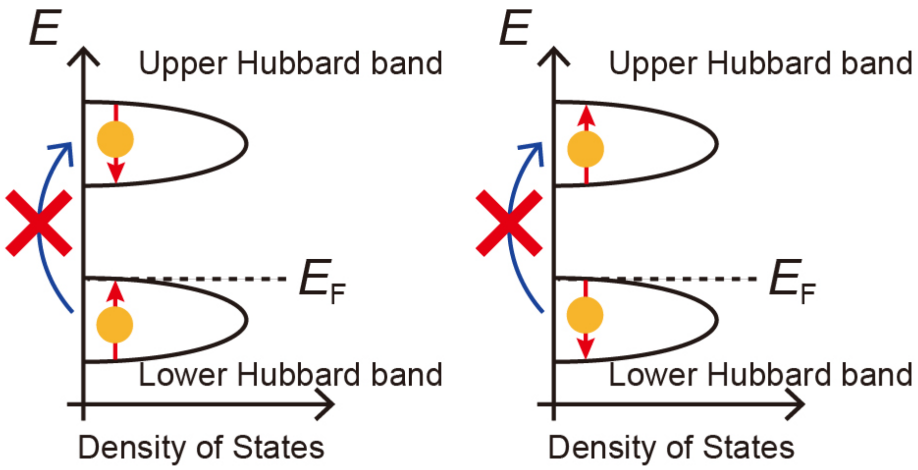

Supplementary Fig. 1| Schematic illustration of the expected mechanism of the suppression of spin scattering in the correlated metal layer of LTO. Spin scattering is expected to be significantly suppressed by the strong on-site Coulomb repulsion in the strongly correlated system. Here, E_F represents the Fermi level.

Supplementary Note 2: Sample characterizations

The atomic force microscopy (AFM) measurement for the $La_{0.67}Sr_{0.33}MnO_3$ (LSMO) [30 unit cells (u.c.) ≈ 12 nm]/LTO (3 u.c. ≈ 1.2 nm) sample grown on a $SrTiO_3$ (STO) substrate shows atomic steps with a period about 200 nm, which is the same as that of the STO surface, indicating that layer-by-layer growth occurs (Supplementary Fig. 2).

As a reference, we also grew a single LTO layer with a thickness of 20 u.c. on STO. The out-of-plane X-ray diffraction (XRD) pattern and reciprocal space mapping for both LSMO/LTO/STO and LTO/STO samples show only pseudo-cubic (00*l*) peaks with Laue (Kiessig) fringes (see the dotted square in Supplementary Fig. 3a), confirming that these samples are single-phase with abrupt interfaces and have no discernible $La_2Ti_2O_7$ phase (Fig. 1e and Supplementary Fig. 3, 4).

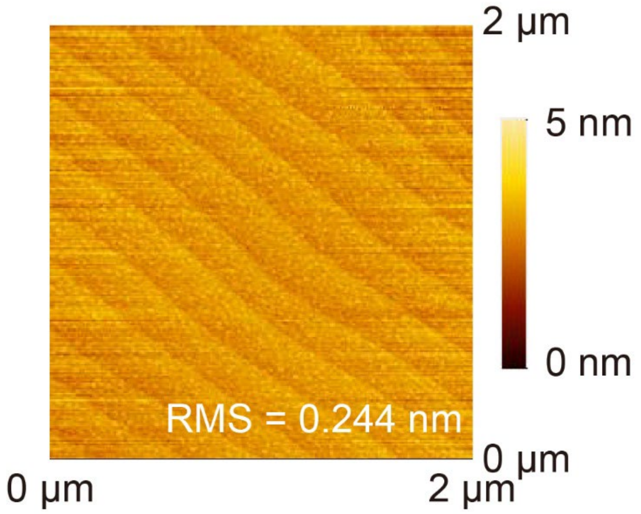

Supplementary Fig. 2| Atomic-force-microscope image of the surface of LSMO/LTO/STO. The root-mean-square roughness (RMS) is 0.244 nm.

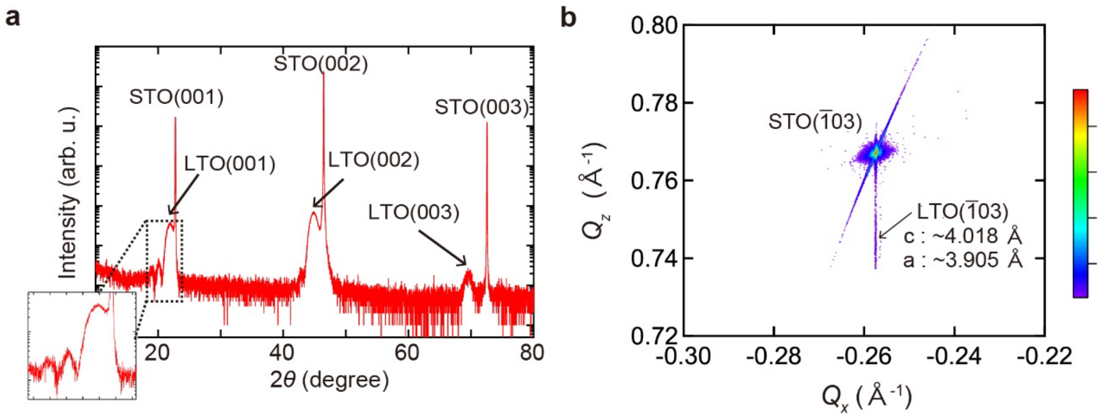

Supplementary Fig. 3| XRD patterns of LTO on the STO substrate. a XRD 2θ-ω scan of LTO (20 u.c.)/STO. In the dotted square region, we see Laue fringes of the LTO (001) peak. **b** Reciprocal space mapping of this sample. Q_z and Q_x represent the components of the reciprocal lattice vector in the perpendicular and in-plane [100] directions, respectively.

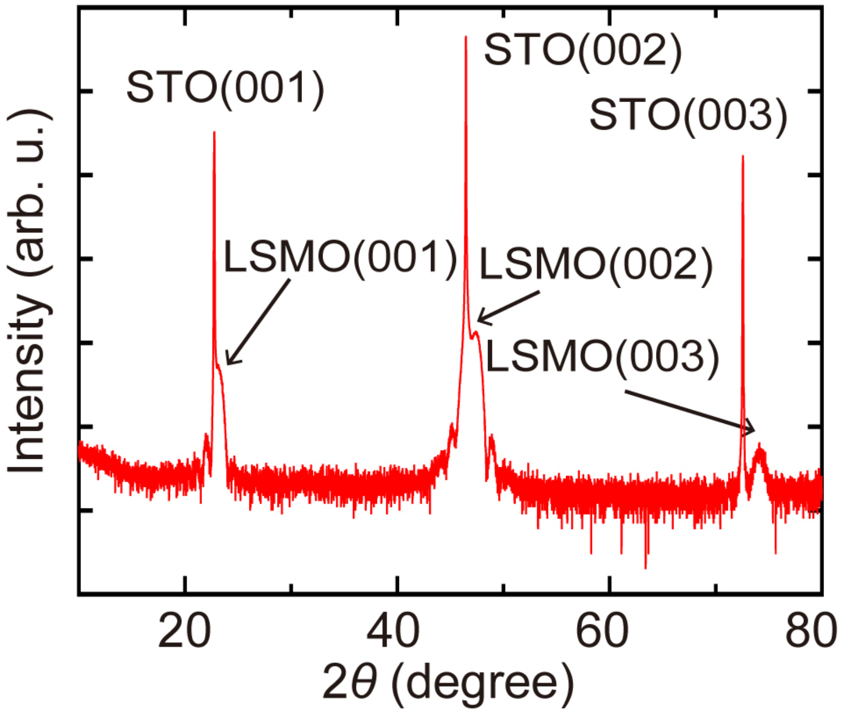

Supplementary Fig. 4| XRD pattern of LSMO (30 u.c.)/LTO (3 u.c.) grown on an STO substrate.

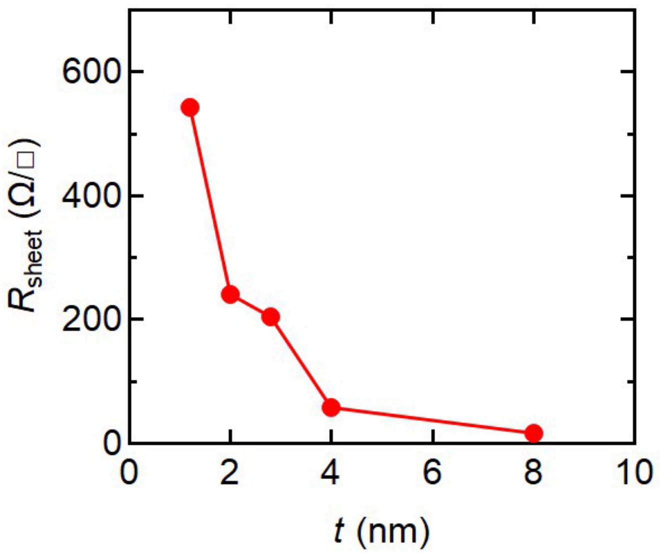

Supplementary Fig. 5| Thickness t dependence of the sheet resistance R_{sheet} for the LTO (t nm) layer grown on STO with a substrate temperature T_{sub} of 720 °C with a low oxygen pressure of 2×10^{-7} Pa. The measurement temperature is 300 K.

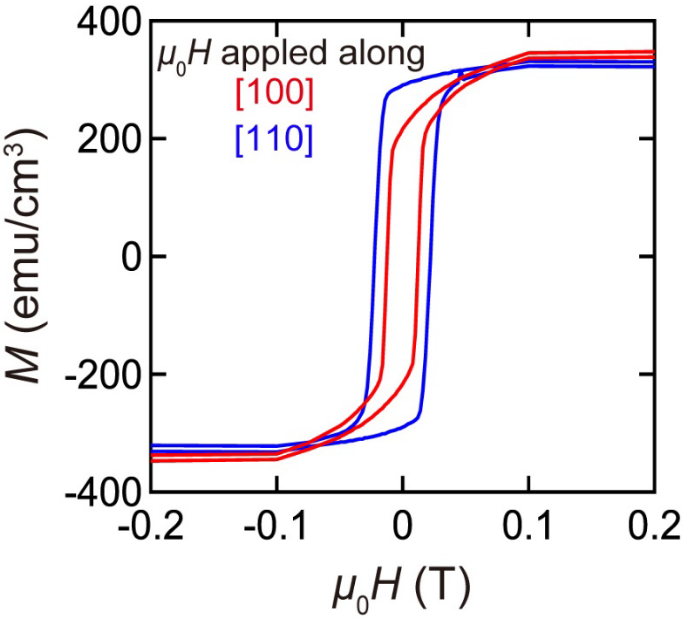

Supplementary Fig. 6| Magnetization *M* measured for LSMO (30 u.c.)/LTO (3 u.c.)/ $(LaAlO_3)_{0.3}(Sr_2TaAlO_6)_{0.7}$ (LSAT) at 10 K with a magnetic field $\mu_0 H$ applied along the [100] and [110] directions of the LSAT substrate.

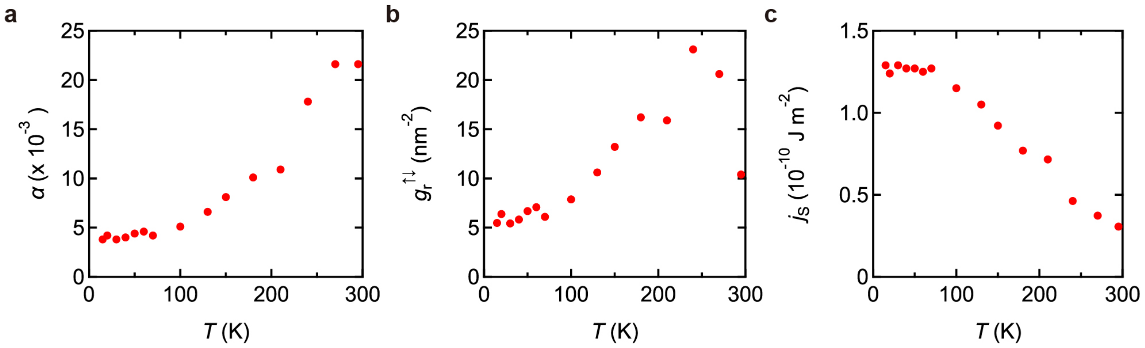

Supplementary Fig. 7| Parameters obtained in our spin pumping measurements. a Damping constant α. **b** Mixing conductance $g_r^{\uparrow\downarrow}$. **c** Spin current j_S.

Supplementary Note 3: Estimation of the relaxation time τ_e of electrons

The relation between the two-dimensional current $j_C{}^{2D}$ and the electric field F in the x direction is expressed by

$$j_C^{2D} = \sum_n \frac{e^2}{4\pi^2\hbar} \int^{FS_n} F\, \tau(|\mathbf{k}|) v_x(\mathbf{k}) \frac{v_x(\mathbf{k})}{|\mathbf{v}(\mathbf{k})|} dS_F\, , (S1)$$

where n is the index of the Fermi surface FS_n, e is the free electron charge, $\hbar$ is the Dirac constant, $v_x(\mathbf{k})$ is the x direction component of the group velocity $\mathbf{v}(\mathbf{k})$, and dS_F is the infinitesimal area (= length in two dimensions) of the Fermi surface. Using Supplementary Eq. (S1) and the experimental sheet resistance of the LTO/STO interface (see Fig. 2a) (here, we neglect the small conductance of LSMO in the LSMO/LTO/STO sample), we can obtain the momentum relaxation time τ_e of electrons by

$$\tau_e = \left[\sum_n \int^{FS_n} \tau(|\mathbf{k}|) dS_F\right] / \left(\sum_n \int^{FS_n} dS_F\right). (S2)$$

The obtained τ_e at each temperature T is shown in Supplementary Fig. 8.

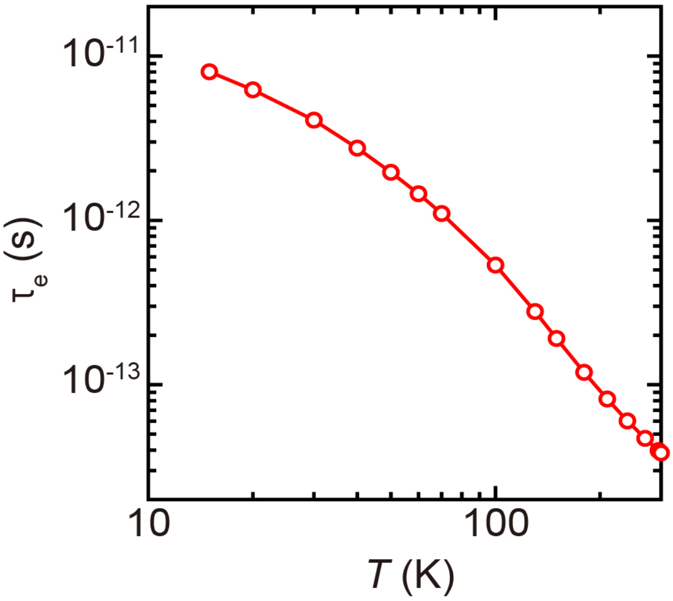

Supplementary Fig. 8| *T* dependence of τ_e.

Supplementary Note 4: Theoretical derivation of the band structure and inverse Edelstein length using the effective tight-binding calculation

We calculated the band structure with the effective tight binding model to reproduce the experimental results of the band structure in Fig. 4a, Fermi surface in Fig. 4b and inverse Edelstein length λ_{IEE} in Fig. 4c (see "Calculation of the λ_{IEE}" in the Methods in the main text). We calculated the density of states (DOS) and the sheet carrier density n_{sheet} at the LTO/STO interface based on the band structure obtained with this model by the following processes:

1. To reproduce the resonant angle-resolved photo emission spectroscopy (R-ARPES) results, we set the parameters Δ_{E1} = 0.172 eV and Δ_{E2} = 0.042 eV in the effective tight-binding model (see each definition in the Methods). In addition, we set Δ_{ASO} and Δ_z as variable parameters so that the experimental λ_{IEE} can be reproduced by the calculation.
2. From the band structure obtained in process 1, we theoretically calculated the Fermi level E_{F} dependence of the DOS and n_{sheet}. We measured the Hall effect in LTO (3 u.c.)/STO, from which we estimated n_{sheet} to be ~8.9×10^{13} cm^{-2} at 4 K. We determined the Fermi level E'_{F} of the LSMO/LTO/STO sample from this n_{sheet} using the calculated E_{F} dependence of n_{sheet} (see Supplementary Fig. 9).
3. We checked whether the theoretical λ_{IEE} using the E'_{F} value obtained above can reproduce the experimental λ_{IEE} or not. If not, we went back to process 1, changed the values of Δ_{ASO} and Δ_z, and followed the process 1 to 3 again.

After repeating the above-mentioned procedure, we found that the conditions of Δ_{ASO} = 0.015 eV and Δ_z = 0.02 eV can well reproduce the experimental λ_{IEE}. In Supplementary Fig. 9, one can see that $E_{\mathrm{F}} = E'_{\mathrm{F}} = -0.102$ eV corresponds to n_{sheet} = ~8.9×10^{13} cm^{-2}.

The differences in n_{sheet} and E_{F} of LTO/STO between the measurement methods, *i.e.* R-ARPES and the Hall measurement, may be due to the difference in oxidization of the sample. For the R-ARPES measurement, the oxidation was suppressed because the sample was transferred under a full nitrogen atmosphere. Meanwhile, for the Hall measurement, the sample was exposed to the atmosphere, and thus the surface was more oxidized. In LSMO/LTO/STO, the LTO layer is thought to be influenced by the large

ozone flux during the growth of the LSMO layer. Thus, we think that the oxidation status of the LTO layer in LSMO/LTO/STO is closer to that of the LTO/STO sample just before the Hall measurement. Thus, we used n_{sheet} and E'_{F} estimated from the Hall effect for the calculation of λ_{IEE}.

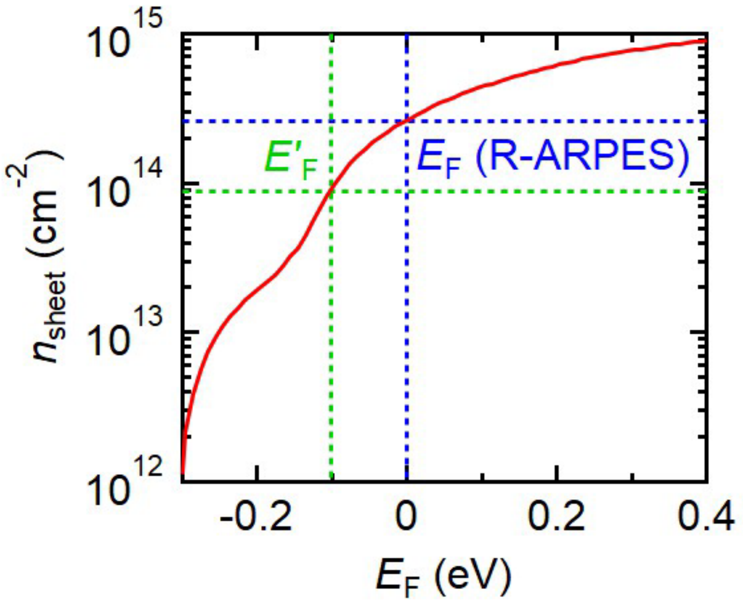

Supplementary Fig. 9| Theoretical calculation of the Fermi level E_{F} dependence of the sheet carrier density n_{sheet}. The crossing points of the dotted lines are the E_{F} and n_{sheet} estimated from the Hall effect (green, $E_{\mathrm{F}} = E'_{\mathrm{F}} = -0.102$ eV) and those estimated from the R-ARPES measurement (blue, $E_{\mathrm{F}} = 0$ eV). From the R-ARPES experiment, the Fermi level is estimated to be located at ~0.3 eV higher than the 1st d_{xy} subband bottom (see Fig. 4a in the main manuscript).

As we mentioned above, we measured the Hall effect for the LTO (3 u.c.)/STO sample, for which the sheet carrier density n_{sheet}, mobility and the sheet resistance R_{sheet} were estimated to be ~8.9×10^{13} cm^{-2}, 6.9×10^{3} cm^{2}/Vs and ~8 Ω/□ at 4 K, respectively. R_{sheet} of ~2.4 Ω/□ obtained for the LSMO/LTO/STO sample at 5 K (see Fig. 2a in the main manuscript, and the possible reason for the difference in R_{sheet} between LSMO/LTO/STO and LTO/STO is noted in Supplementary Note 4) is relatively low compared with other two-dimensional electron gas (2DEG) systems formed at STO interfaces. However, this R_{sheet} value is well within the range of the reported values as shown in Supplementary Fig. 10, in which we summarize R_{sheet} vs. n_{sheet} reported for

2DEGs at various STO interfaces grown by sputtering, pulse laser deposition (PLD) and molecular beam epitaxy (MBE). As indicated by the green points obtained for $LaAlO_3$ (LAO)/STO, in comparison with the samples grown by PLD (see the open green points), lower R_{sheet} can be realized by using MBE (see the filled green points). This trend is probably due to the suppression of interface roughness scattering. We note that the 2DEG at the γ-Al_2O_3 (GAO)/STO interface has a low R_{sheet} of ~1.5 Ω/□ with n_{sheet} of ~8.5×10^{13} cm^{-2}, even though PLD was used for the growth (see the blue open points)[7]. The light orange area in Supplementary Fig. 10 expresses the range of R_{sheet} and n_{sheet} reported for various STO interfaces. Our data of R_{sheet} of ~2.4–8 Ω/□ with n_{sheet} ~8.9×10^{13} cm^{-2} obtained in the LSMO/LTO/STO and LTO/STO heterostructures (see the dark orange region) are well within this range.

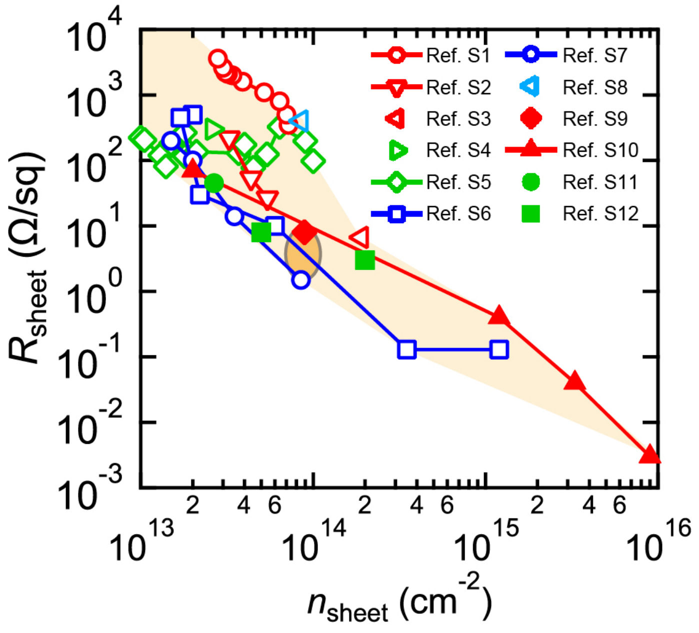

Supplementary Fig. 10| R_{sheet} vs. n_{sheet} at various STO interfaces. The red, green, blue and light blue points correspond to the data of LTO/STO, LAO/STO, GAO/STO and AlO_x/STO, respectively. The filled and open points are the data of samples made by MBE and PLD, respectively (only the light blue point corresponds to a sample made by sputtering). The light orange area is the range of R_{sheet} and n_{sheet} for various STO interfaces reported thus far[S1–S12]. The dark orange area is the range of R_{sheet} obtained in our LSMO/LTO/STO and LTO/STO heterostructures.

Supplementary Note 5: E_F dependence of calculated λ_{IEE} and Fermi surfaces

Supplementary Fig. 11 shows the E_F dependence of calculated λ_{IEE} and Fermi surfaces with the spin direction at each **k** point for various E_F. Here, we explain how this complicated λ_{IEE} behavior as a function of E_F is determined by the change in the Fermi surfaces, using the region between points 1 and 4 as an example. λ_{IEE} has a maximum value at around point 3, and it takes a local minimum between points 3 and 4 (see Supplementary Fig. 11a). In the energy region between points 1 and 2, the two pairs of the Fermi surfaces have the same spin chirality (see Supplementary Fig. 11c,h; when we closely look at the spin directions at $k_y = 0$ when $k_x > 0$ in Supplementary Fig. 11h, we can see that the outer band is blue and the inner band is red for each pair). Thus, both pairs contribute to the IEE positively, and λ_{IEE} increases with increasing E_F up to point 2. At around point 3, a new pair of Fermi surfaces appears near the center of the k_x–k_y plane (see Supplementary Fig. 11d,i). Its spin chirality is opposite to that of the other two pairs of the Fermi surfaces (closely look at the most central pair of the Fermi surfaces in Supplementary Fig. 11d,i). Thus, as the influence of this most central pair becomes dominant by increasing E_F from point 3, λ_{IEE} decreases towards the local minimum between points 3 and 4. In this way, the magnitude of the IEE is determined mainly by the spin chirality of the Fermi surfaces.

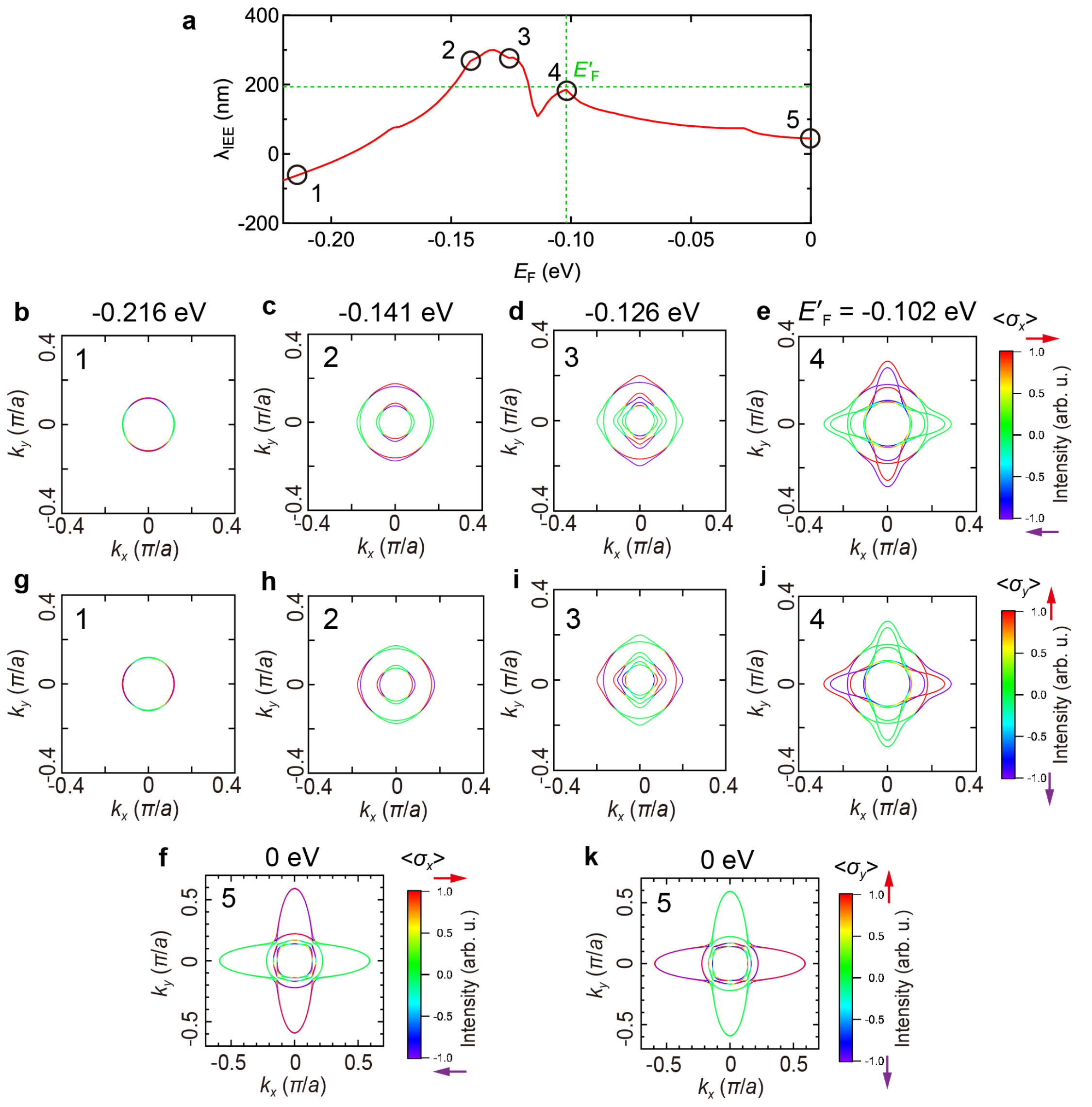

Supplementary Fig. 11| E_F dependence of calculated λ_{IEE} and Fermi surfaces mapped with the spin expectation values of $<\sigma_x>$ and $<\sigma_y>$. a E_F dependence of calculated λ_{IEE}. The crossing point of the dotted lines is the λ_{IEE} and estimated E_F (= E'_F = –0.102 eV) in LSMO/LTO/STO used for the spin pumping measurements. The circles correspond to the E_F positions of the Fermi surfaces shown in **b-k**. λ_{IEE} strongly depends on the E_F due to the spin chirality of each band. **b-k** E_F dependence of the Fermi surface with the spin directions: Fermi surfaces with the spin components in the x direction (**b-f**) and y direction (**g-k**) at E_F = –0.216, –0.141, –0.126, –0.102 and 0 eV. $<\sigma_x>$ and $<\sigma_y>$ are the spin expectation values in the x and y directions.

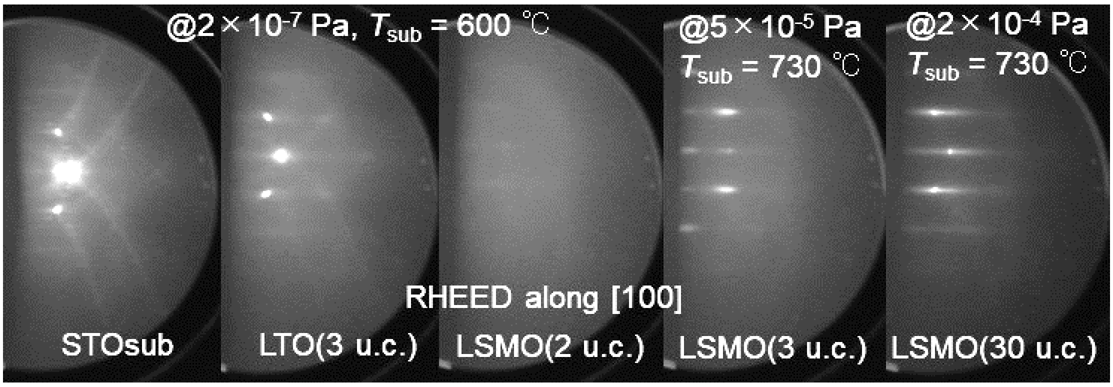

Supplementary Fig. 12| Reflection high-energy electron diffraction (RHEED) patterns during the growth of LTO and LSMO.

Supplementary References

S1. Biscaras, J. et al. Limit of the electrostatic doping in two-dimensional electron gases of $LaXO_3$(X = Al, Ti)/$SrTiO_3$. *Sci. Rep.* **4,** 6788 (2014).

S2. Lee, J. N., Hou, X., Takahashi, R. & Lippmaa, M. Tuning the carrier density in $SrTiO_3$/$LaTiO_3$/$SrTiO_3$ quantum wells. *Appl. Phys. Lett.* **116,** 171601 (2020).

S3. Veit, M. J., Arras, R., Ramshaw, B. J., Pentcheva, R. & Suzuki, Y. Nonzero Berry phase in quantum oscillations from giant Rashba-type spin splitting in $LaTiO_3$/$SrTiO_3$ heterostructures. *Nat. Commun.* **9,** 1458 (2018).

S4. Lesne, E. et al. Highly efficient and tunable spin-to-charge conversion through Rashba coupling at oxide interfaces. *Nat. Mater.* **15,** 1261 (2016).

S5. Chen, Z. et al. Dual-Gate Modulation of Carrier Density and Disorder in an Oxide Two-Dimensional Electron System. *Nano. Lett.* **16,** 6130 (2016).

S6. Chen, Y. Z. et al. A high-mobility two-dimensional electron gas at the spinel/perovskite interface of γ-Al_2O_3/$SrTiO_3$. *Nat. Commun.* **4,** 1371 (2013).

S7. Christensen, D. V. et al. *Phys. Rev. Appl.* **9,** 054004 (2018).

S8. Vaz, D. C. et al. Mapping spin-charge conversion to the band strcture in a topological oxide two-dimensional electron gas. *Nat. Mater.* **15,** 1187 (2019).

S9. Kaneta-Takada, S. et al. in this manuscript.

S10. Edmondson, B. I. et al. Effect of $SrTiO_3$ oxygen vacancies on the conductivity of $LaTiO_3$/$SrTiO_3$ heterostructures. *J. Appl. Phys.* **124,** 185303 (2018).

S11. Sun, H. Y. et al. Chemically specific termination control of oxide interfaces via layer-by-layer mean inner potential engineering. *Nat. Commun.* **9,** 2965 (2018).

S12. Ohya, S. et al. Efficient intrinsic spin-to-charge current conversion in an all-epitaxial single-crystal perovskite-oxide heterostructure of $La_{0.67}Sr_{0.33}MnO_3$/$LaAlO_3$/$SrTiO_3$. *Phys. Rev. Res.* **2,** 012014 (R) (2020).
